# The Proportion of Regulatory T Cells in Patients with Rheumatoid Arthritis: A Meta-Analysis

**DOI:** 10.1371/journal.pone.0162306

**Published:** 2016-09-13

**Authors:** Takayoshi Morita, Yoshihito Shima, James Badger Wing, Shimon Sakaguchi, Atsushi Ogata, Atsushi Kumanogoh

**Affiliations:** 1 Department of Respiratory Medicine, Allergy and Rheumatic diseases, Osaka University Graduate School of Medicine, Osaka, Japan; 2 Department of Experimental Immunology, Immunology Frontier Research Center, Osaka University, Osaka, Japan; 3 Department of Experimental Pathology, Institute for Frontier Medical Sciences, Kyoto University, Kyoto, Japan; 4 Division of Allergy, Rheumatology and Connective tissue disease, NTT west Osaka Hospital, Osaka, Japan; Universite Paris-Sud, FRANCE

## Abstract

**Background:**

Regulatory T cells (Tregs) have important functions in peripheral immune tolerance. Dysfunction of Tregs is considered to be a pivotal cause of autoimmune diseases, including rheumatoid arthritis (RA). However, previous reports describing the proportion of Tregs among CD4^+^ T cells in RA patients were controversial because a range of markers are used to identify Tregs with little consensus. To clarify the status of Tregs in RA, we investigated the proportion of Tregs with focusing on the definitions of them.

**Methods:**

We identified the studies reporting the proportion of Tregs in RA patients using PubMed and Google Scholar. We performed a systematic review of them and a meta-analysis to evaluate the proportion of Tregs (FOXP3-positive and/or CD25-positive) among CD4^+^ T cells in peripheral blood (PB) and synovial fluid (SF) of RA patients and control subjects.

**Results:**

A total 31 studies were selected. The proportion of Tregs defined by all definitions among CD4^+^ T cells in PB was not significantly different between RA patients and control subjects (-0.65, [-1.30, 0.01]). Then we performed sub-analyses based on individual definitions. The proportion of Tregs defined by either CD25 or FOXP3 alone did not differ between RA patients and control subjects. The proportion of Tregs defined by both FOXP3 and CD25 was lower in RA patients than that in control subjects (-2.42 [-3.49, -1.34]). The proportion of Tregs defined by both FOXP3 and CD25 was higher in SF than that in PB among RA patients (3.27 [0.40, 6.14]).

**Conclusion:**

The status of Tregs varied according to the definition system. The proportion of Tregs defined by stricter and functionally validated methods decreased in PB and increased in SF among RA patients. If the proportion of Tregs differs in RA, accurate and functionally relevant definitions of Tregs are necessary to elucidate their status in RA.

## Introduction

Rheumatoid arthritis (RA) is a typical disease of chronic inflammation resulting from impaired immune homeostasis [1]. Clinical studies have shown that inflammatory cytokines such as tumor necrosis factor (TNF)-α and interleukin (IL)-6 are present at high levels in the sera of RA patients and play an important role in RA pathogenesis [2]. In addition, effector T cells such as the T helper17 (Th17) subset of CD4^+^ T cells producing IL-17 have been found to play important roles in the pathology of RA [3]. Furthermore, auto-reactive Th17 cells contribute to arthritogenesis in the SKG model of mouse arthritis [4]. In contrast, specialized regulatory cells suppress autoimmune response and maintain immune tolerance. Regulatory T cells (Tregs) are a distinct set of thymically produced T cells responsible for suppressing autoreactive deleterious activities of effector T cells [5]. The forkhead box P3 (FOXP3) transcription factor is considered the most reliable marker for Tregs and is essential to their regulatory function [6]. Dysfunction of *FOXP3* is the cause of IPEX (immune dysregulation, polyendocrinopathy, enteropathy, and X-linked) syndrome, which is associated with refractory diarrhea, type1 diabetes, hypothyroidism, and glomerulonephritis [6]. IPEX is a fulminant autoimmune disease resulting from complete failure of self-tolerance. Failure of suppressive functions of Tregs is a possible cause of arthritogenesis as arthritis has also been observed in IPEX patients [7]. Consequently, the balance of Tregs and effector cells is critical to the maintenance of the self-tolerance.

Recently, the significance of Tregs in RA pathology has been a focus of much research. In the collagen induced arthritis (CIA) mouse model, reduction of Tregs and induction of Th17 cells has been reported [8]. In this model, a reduction in the proportion of Tregs following treatment with anti-CD25 antibody exacerbates arthritis [9], whereas injection of Tregs ameliorates the symptoms [10]. In the SKG model, arthritis is exacerbated by a reduction of FOXP3 function [4]. In human, *FOXP3* genetic polymorphism and the FOXP3 protein level may be associated with susceptibility to RA [11]. Polymorphisms of cytotoxic T-lymphocyte associated antigen 4 (CTLA-4), one of the main suppressive molecules in Tregs [12], have been reported in RA patients and are associated with RA risk and reduced suppressive functions of Tregs [13]. Meanwhile, inflammatory cytokines such as TNF-α and IL-6 ameliorates the function of Tregs. TNF-α suppresses the expression of FOXP3 [14], while IL-6 suppresses the induction of Tregs from naïve T cells and promotes the induction of Th17 cells, thereby exacerbating RA pathology [15]. Although the results of basic studies strongly suggest that Tregs in RA play a role in the pathology of RA, the function of Tregs in peripheral immune tolerance of RA patients remains controversial.

The proportion of Tregs among CD4^+^ T cells in peripheral blood (PB) is considered a reliable method for the estimation of Tregs function in peripheral immune tolerance. Molecular markers are essential tools for identifying Tregs. Since Tregs express high levels of CD25, the alpha-chain of the IL-2 receptor, Tregs were first defined as CD25-positive cells [16]. Further to this, CTLA4, CD25, FOXP3, and loss of CD127 are widely used as markers for Tregs. However, these markers are not truly specific for Tregs as activated non-Tregs may also upregulate their expression, making it difficult to distinguish Tregs from activated effector T cells [17, 18]. As a result, reports of the status of Tregs in humans must be subjected to careful analysis.

In this study, we systemically reviewed original studies in which authors documented the proportion of Tregs among CD4^+^ T cells in PB or SF of RA patients, and performed a meta-analysis to evaluate the proportion of Tregs in RA patients with focusing on definition of Tregs.

## Materials and Methods

### Data sources and searches

We searched for studies documenting the proportion of Tregs among CD4^+^ T cells in PB of RA patients using two search websites, PubMed and Google Scholar. On PubMed, searches identified publications with articles containing the term “rheumatoid arthritis,” (“regulatory T,” or “Treg,”) and “human and patient”, but not containing the term “review or mice or rat” (Indeed, we performed searching by [“rheumatoid arthritis" AND ("regulatory T" OR Treg) AND (human OR patient) NOT (review OR mice OR rat)]. On Google Scholar, searches identified publications with titles containing the term “rheumatoid arthritis” and “regulatory T,” “Treg,” “CD25,” or “FOXP3” (Indeed, we performed searching by [allintitle:"rheumatoid arthritis" ("regulatory T" OR "Treg" OR "CD25" OR "Foxp3")]. Searches were aimed at identifying studies published between January 1, 2005 and May 31, 2016. We searched for studies four times using the same terms and the same websites. Final searches were performed on June 18, 2016.

### Study selection

Inclusion criteria: 1) original studies (not reviews); 2) human research; 3) title or abstract including the terms “rheumatoid arthritis” and “regulatory T” (or “Treg”); 4) studies reporting the proportion of Tregs among CD4^+^ T cells in PB of RA patients; 5) available on the internet; the manuscript is linked from search site to the full text (PDF or website) of the manuscript. Simultaneously, we verified that all RA patients in the selected studies were diagnosed according to the American college of rheumatology (ACR) criteria from 1987 or the ACR/ The European League Against Rheumatism (EULAR) criteria from 2010.

Exclusion criteria: 1) studies that provided no raw data regarding the average and standard deviation (SD) of the proportion of Tregs among CD4^+^ T cells in PB of RA patients or control subjects; 2) studies that provided no raw data about the number of RA patients or control subjects. Redundancies between PubMed and Google Scholar search were eliminated, i.e., individual studies were counted only once in the analysis.

### Validity and quality assessment

Two authors independently checked and selected all references. We assessed the evidence level of selected studies based on guidelines from the Oxford Center for Evidence-Based Medicine 2011 [19]. We validated and performed a quality assessment of our systematic review based on the Newcastle–Ottawa Quality Assessment Scale (Case Control Studies) [20]; the Newcastle-Ottawa Scale is a tool for assessing the quality of non-randomized studies in meta-analyses.

### Data extraction

We extracted the following data from all assessed studies: author, country, year of publication, number of RA patients and control subjects (including healthy persons and patients with osteoarthritis [OA]), mean (or median) and SD of the proportion of Tregs among CD4^+^ T cells in PB of RA patients and control subjects, and definition of Tregs. When Tregs were defined by several patterns such as CD25-positive, CD25-high, FOXP3-positive, CD25^+^ CD127-low, and/or CD25^+^ FOXP3^+^ in a study, we extracted all types of data. When a study reported the proportion of Tregs among CD4^+^ T cells in PB of both active (acute) RA and remission (late phase) RA, we extracted both types of data. When a study reported the proportion of Tregs among CD4^+^ T cells in synovial fluid (SF), we extracted the SF data as well. In addition, we extracted the background data of RA patients (age, proportion of female subjects, disease duration, positive rate of rheumatoid factor [RF], disease activity score 28 [DAS28], C reactive protein [CRP] level, erythrocyte sedimentation rate [ESR], and treatment information).

### Quantitative data synthesis

We performed quantitative data synthesis based on the Preferred Reporting Items for Systematic Reviews and Meta-Analyses (PRISMA) Statement [21] (S1 File). We performed a meta-analysis to provide quantitative summary estimates of the standardized mean difference (SMD); the proportion of Tregs among CD4^+^ T cells in PB of RA patients minus that of control subjects, the proportion of Tregs among CD4^+^ T cells in PB of active RA patients minus that of remission RA patients, or the proportion of Tregs among CD4^+^ T cells in SF of RA patients minus that in PB of RA patients. Summary SMD was calculated using a random-effects model (REM) (DerSimonian and Laird method) [22]. REM was performed in our analysis because there were clear differences in the experimental methods or techniques among selected studies. When we evaluated publication bias in all selected studies by funnel plot, summary averages were also calculated using a fixed-effects model according to Peto and Mantel-Haenszel. Statistical significance was defined as P < 0.05. The I^2^ statistic was calculated to assess statistical heterogeneity across studies [23]. For I^2^ values >50%, we explored characteristics of individual studies and subgroups of the main body of evidence. All analyses were conducted using R version 3.1.2 (R project for Statistical Computing) and EZR version 1.29 [24].

## Results

### Study characteristics

**Fig 1:**
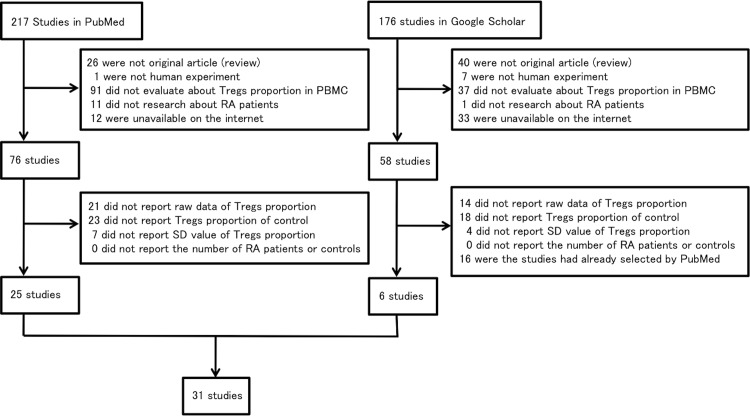
Flow diagram of study selection.

We found 217 studies on PubMed by the search terms. Of these, 141 were not selected because they did not satisfy the inclusion criteria, and 51 were excluded because they satisfied the exclusion criteria. Ultimately, we selected 25 studies from PubMed. Similarly, we found 176 studies on Google Scholar by the search terms. Of these, 118 studies were not selected because they did not satisfy the inclusion criteria, and 36 were excluded because they satisfied the exclusion criteria. 16 were eliminated because they had already been selected by the PubMed search. Ultimately, we selected six studies from Google Scholar. In total, 31 studies were used for our analysis (Fig 1, Table 1) [25–55]. Study (a), (d), (i), (o), (r), (s), (u), (v) and (y) were found on PubMed only, (b), (c), (l), (w), (aa), and (ad) were found on Google Scholar only, and the others were found on both PubMed and Google scholar.

**Table 1:** Study information about proportion of Tregs in RA patients or control subjects

Author (Study identifier) [Ref.]	Publish Year	EL	Q	Country	Case Numbers	Tregs definition	% of Tregs among CD4^+^ T cells [mean (or median) ± SD]
(a) Alessandro B. et al. [25]	2015	4	3	Italy	RA: 14, HC: 8	CD25+ FOXP3+	RA: 5 ± 3.8, HC: 4.5 ± 1.8
(b) Al-Zifzafa DS. et al. [26]	2015	4	7	Egypt	RA: 40, HC: 20	CD25high FOXP3+	RA: 16.9 ± 6.2, HC: 26.4 ± 14.0
(c) Gaafar T. et al. [27]	2015	4	7	Egypt	RA: 38, HC: 50	CD25+ FOXP3	RA: 1.01 ± 0.87, HC: 1.72 ± 1.27
(d) Daïen CI. et al. [28]	2015	4	6	France	RA: 92, HC: 25	CD25high CD127-	RA: 1.5 ± 0.9, HC: 2.1 ± 0.9
(e) Cribbs AP. et al. [29]	2014	4	6	UK	RA: 18, HC: 10	CD25+ CD127-	RA: 6.15 ± 2.4, HC: 4.76 ± 0.9
(f) Moradi B. et al. [30]	2014	4	5	Germany	RA: 18, OA: 22	CD25high CD25+ CD127-	RA: 4.88 ± 2.04, OA: 4.4 ± 1.59 RA: 6.7 ± 1.8, OA: 7.6 ± 1.8
(g) Matsuki F. et al. [31]	2014	4	5	Japan	RA: 32, HC: 19	CD45RA- FOXP3high	RA: 1.0 ± 0.9, HC: 2.1 ± 1.8
(h) Guggino G. et al. [32]	2014	4	7	Italy	RA: 15, HC: 10	CD25+ IL10+ IL17-	RA: 1.0 ± 0.03, HC: 2.3 ± 0.6
(i) Ji L. et al. [33]	2014	4	7	China	RA: 21, HC: 8	FOXP3+	RA: 1.91 ± 0.77, HC: 1.78 ± 1.55
(j) Gao S. et al. [34]	2014	4	5	China	RA: 35, HC: 17	CD25+ FOXP3+	RA: 2.89 ± 0.17, HC: 5.83 ± 0.39
(k) Nie H. et al. [35]	2013	4	7	China	RA: 20, HC: 20	CD25high	RA: 1.7 ± 0.88, HC: 1.7 ± 0.86
(l) Abazaa N. et al. [36]	2013	4	7	Egypt	RA: 20, HC: 20	CD25+	RA: 19.7 ± 0.57, HC: 14.4 ± 0.56
(m) Kim JR. et al. [37]	2012	4	6	Korea	RA: 42, HC: 22	CD45RA- CD25high CD25high FOXP3+	RA: 0.4 ± 0.2, HC: 0.6 ± 0.3 RA: 3.8 ± 1.0, HC: 5.0 ± 1.3
(n) Niu Q. et al. [38]	2012	4	6	China	RA: 36, HC: 20	CD25+ FOXP3+	RA: 1.54 ± 0.23, HC: 4.33 ± 0.35
(o) Chen J. et al. [39]	2012	4	4	China	RA: 76, HC: 18	CD25+ FOXP3+	RA: 1.8 ± 1.2, HC: 2.6 ± 1.8
(p) Chen R. et al. [40]	2012	4	-	China	RA: 40, HC: 40	CD25+ FOXP3+	RA: 5.36 ± 1.55, HC: 7.49 ± 1.46
(q) Xiao H. et al. [41]	2011	4	6	China	RA: 35, HC: 27	CD25+ FOXP3+	RA: 3.72 ± 0.83, HC: 5.24 ± 1.05
(r) Furuzawa-Carballeda J. et al. [42]	2011	4	7	Mexico	RA: 14, HC: 11	FOXP3+	RA: 6.1 ± 0.9, HC: 4.2 ± 0.6
(s) Loza MJ. et al. [43]	2011	4	5	USA	RA: 6, HC: 24	FOXP3+	RA: 3.5 ± 1.0, HC: 3.8 ± 1.5
(t) Tang TT. et al. [44]	2011	4	5	China	RA: 55, HC: 42	CD25+ FOXP3+	RA: 5.2 ± 1.3, HC: 5.0 ± 1.5
(u) Chen MH. et al. [45]	2011	4	4	China	RA: 20, HC: 25	CD25high	RA: 1.95 ± 0.17, HC: 2.16 ± 0.10
(v) Ursaciuc C. et al. [46]	2010	4	4	Romania	RA: 7, HC: 10	CD25+	RA: 4.2 ± 2.5, HC: 4.7 ± 1.2
(w) Al-Shukaili A. et al. [47]	2009	4	5	Oman	RA: 10, HC: 20	CD25bright	RA: 0.56 ± 0.29, HC: 1.74 ± 0.47
(x) Sempere-Ortells JM. et al. [48]	2009	4	7	Spain	RA: 60, HC: 40	CD25+	RA: 6.8 ± 0.4, HC: 10.7 ± 1.1
(y) Huang ZX. et al. [49]	2009	4	-	China	RA: 25, HC: 31	CD25+ CD127- CD25high CD127-	RA: 2.53 ± 0.85, HC: 3.22 ± 0.97 RA: 0.91 ± 0.32, HC: 1.25 ± 0.41
(z) Han GM. et al. [50]	2008	4	7	USA	RA: 99, HC: 44	CD25high	RA: 23 ± 1.3, HC: 14.4 ± 0.8
(aa) Yoon BY. et al. [51]	2007	4	6	Korea	RA: 12, HC: 10	CD25+	RA: 16.1 ± 4.2, HC: 6.13 ± 3.5
(ab) Kao JK. et al. [52]	2007	4	7	Taiwan	RA: 19, HC: 9	CD25+ CD25high	RA: 15.78 ± 10.04, HC: 4.90 ± 3.45 RA: 1.49 ± 1.57, HC: 0.2 ± 0.25
(ac) Jiao Z. et al. [53]	2007	4	7	China	RA: 11, HC: 11	CD25+ CD25high CD25+ FOXP3+	RA: 10.34 ± 3.4, HC: 11.4 ± 3.7 RA: 2.3 ± 0.9, HC: 4.1 ± 1.5 RA: 3.1 ± 1.5, HC: 5.5 ± 1.2
(ad) Minami R. et al. [54]	2006	4	3	Japan	RA: 101, HC: 12	CD25high	RA: 2.7 ± 1.42, HC: 4.05 ± 1.95
(ae) Möttönen M. et al. [55]	2005	4	6	Finland	RA: 10, HC: 9	CD25+	RA: 5.5 ± 4.1, HC: 6.7 ± 5.0

Evidence level (EL) of each study was based on Oxford Center for Evidence-Based Medicine 2011 [19]. Quality (Q) of each study was based on Newcastle Ottawa Quality Assessment Scale Case [20]. (p) and (y) were not evaluated Quality score by The New Castle-Ottawa Assessment Scale in detail because these manuscripts were written in Chinese.

We provide the details of the selected studies in S1 Table. We extracted clinical data regarding RA patients; ranges were as follows: average age, 33–68 years; proportion of female subjects, 17–100%; disease duration, 0.8–16 years; average CRP value, 0.2–24.0 mg/dl; average ESR, 20–77 mm/hour; DAS28, 3.1–7.0; positive ratio of RF, 56–90%. Patients described in the studies were treated with disease-modifying anti-rheumatic-drugs (DMARDs), methotrexate (MTX), corticosteroids (CS), or biological agents such as TNF-α inhibitor (iTNF) and IL-6 receptor inhibitor (tocilizumab; TCZ). Most control subjects were healthy individuals, but OA patients were selected as controls in one study that evaluated SF. The definition of Tregs was various (Table 1) (25–55). The proportion of Tregs among CD4^+^ T cells was 0.4–23.0% in PB of RA patients and 0.20–26.4% in PB of control subjects. The studies reported data from 6–101 RA subjects and 8–44 control subjects. The selected studies were published by groups in USA, UK, France, Germany, Italy, Spain, Finland, Japan, Korea, China, Romania, Taiwan, Mexico, Oman, and Egypt. All studies were evidence level 4 (poor-quality case-control studies or case series). Although not all selected studies were case-control studies per se (e.g., some included bench research), we regarded them as such and scored them by The Newcastle Ottawa Quality Assessment Scale (NOQAS). The NOQAS score was 3–7 for all studies (S2 Table).

Publication bias was evaluated utilizing a funnel plot (S1 Fig), which indicated that each study was located apparently symmetrically.

### The proportion of Tregs in peripheral blood of RA patients

**Fig 2:**
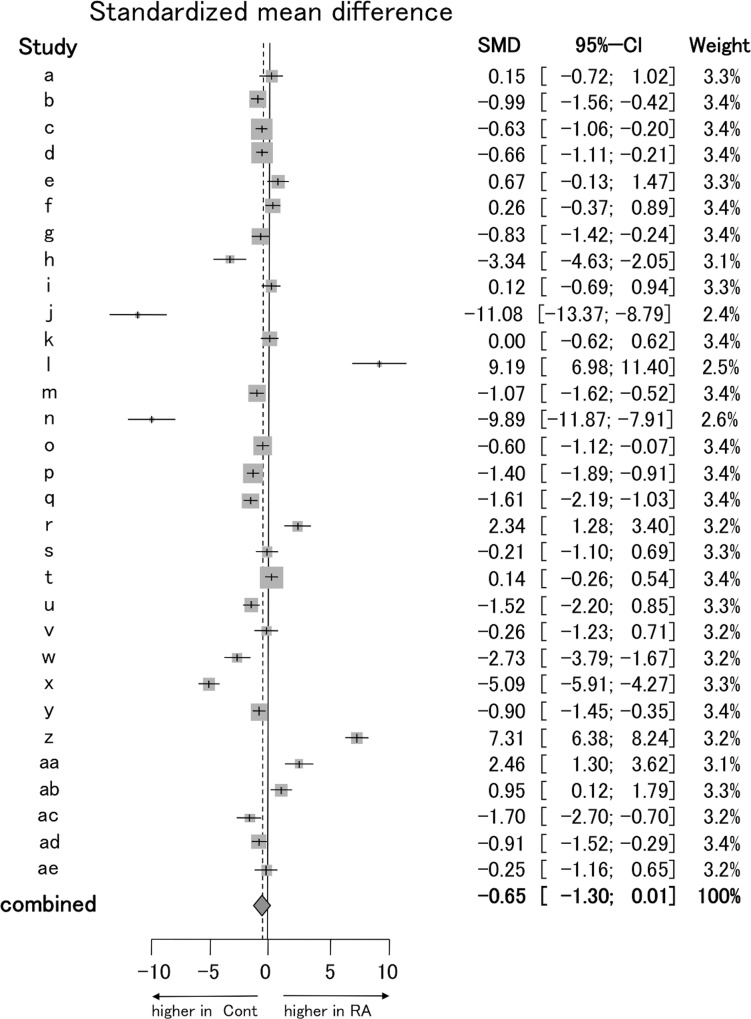
Forest plot for combined meta-analysis of the proportion of Tregs in peripheral blood. Standardized mean difference (the proportion of Tregs among CD4^+^ T cells in PB of RA patients minus that of control subjects) was estimated by meta-analysis. All definitions of Tregs were selected for analysis. Studies distinguished by study identifier (a)–(ae) (Table 1).

At first, we performed meta-analysis of the proportion of Tregs between RA patients and control subjects regardless of the definition of Tregs (Fig 2). There was no significant difference in the proportion of Tregs among CD4^+^ T cells in PB between RA patients and control subjects in all studies (a)–(ae) (REM -0.65, [95% CI: (-1.30 to 0.01)]). Surprisingly, some studies showed quite different results. Some studies showed that the proportion of Tregs was higher in controls (b, h, j, m, n, p, q, u, w, x, ac). Other studies showed that the proportion of Tregs was higher in RA (l, r, z, aa, ab). Heterogeneity assessed by I^2^ statics was 96.3% (P < 0.0001) indicated extreme heterogeneity.

**Fig 3:**
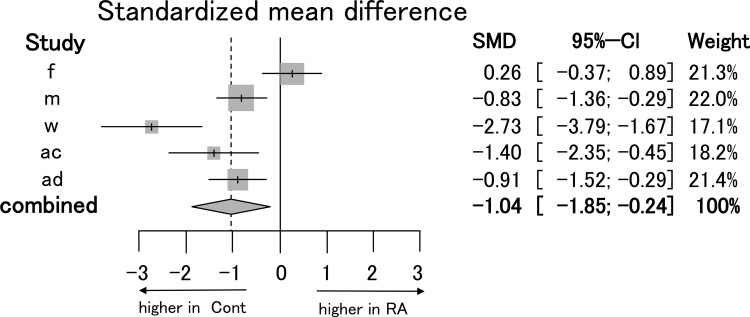
Forest plot for sub-group meta-analysis of the proportion of Tregs defined by CD25-high. Standardized mean difference (the proportion of Tregs among CD4^+^ T cells in PB of RA patients minus that of control subjects) was estimated by meta-analysis. Tregs, which were defined by “CD25-high”, were selected for sub-analysis.

We hypothesized that the cause of significant dispersion may be different definitions of Tregs. Then we performed subgroup analysis based on individual definitions. First, we analyzed the studies (l, v, x, aa, ab, ac, ae) in which “CD25-positive alone” was used to define Tregs (S2A Fig). Because the definition of Tregs in study h was IL-10^+^, IL17^-^, and CD25^+^, study h was removed from this sub-analysis. There was no significant difference in the proportion of Tregs among CD4^+^ T cells in PB between RA patients and control subjects (REM 0.88, [-1.46 to 3.21]) (I^2^ = 97.4%, P < 0.0001). Next, we also analyzed the studies (f, m, w, ac, ad) in which “CD25-high” was used to define Tregs (Fig 3). Some studies (k, u, z, ab) were removed from this sub-analysis because CD25-high gating strategy was not clearly demonstrated. The cutoff line defining CD25-high was shown and either clearly higher than non-CD4 cells (f, w, ad), higher than CD45RA^+^CD25lo Naïve Tregs and CD45RA-CD25lo non-Tregs (m) or CD25 cells were demonstrated to be mostly FOXP3^+^ (ac). There was a significant reduction in the proportion of Tregs, when they were defined by “CD25-high” among CD4^+^ T cells in PB between RA patients and control subjects (REM -1.04, [-1.85 to -0.24]) (I^2^ = 84.2%, P < 0.0001).

Tregs defined as “CD25-positive and CD127-negative” (e, f, y) showed no significant difference in the proportion of Tregs among CD4^+^ T cells in PB between RA patients and control subjects (REM -0.23, [-1.01 to 0.54]) (S2B Fig). Heterogeneity assessed by I^2^ statics was 76.4% (P = 0.0144), suggesting moderate heterogeneity.

**Fig 4:**
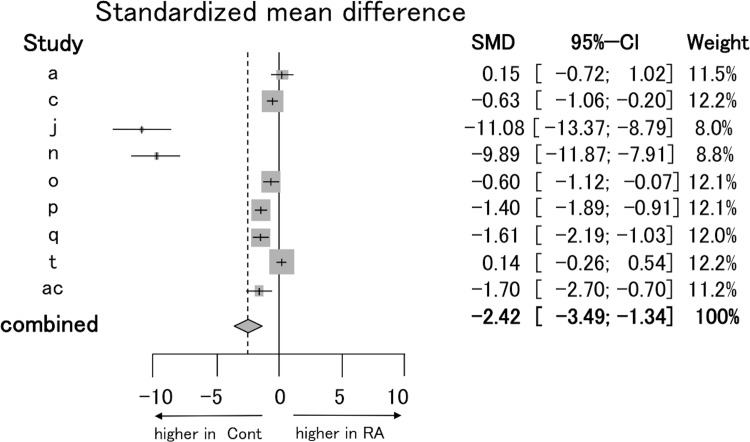
Forest plot for sub-group meta-analysis of the proportion of Tregs defined by CD25 and FOXP3 double positive. Standardized mean difference (the proportion of Tregs among CD4^+^ T cells in PB of RA patients minus that of control subjects) was estimated by meta-analysis. Tregs, which were defined by “CD25 and FOXP3 double positive”, were selected for sub-analysis.

Then, we analyzed the studies (i, r, s) in which Tregs were defined as “FOXP3-positive alone” (S2C Fig). There was no significant difference in the proportion of Tregs defined by “FOXP3-positive alone” in PB between RA patients and control subjects (REM 0.72, [-0.72 to 2.16]) (I^2^ = 86.5%, P = 0.0006). The proportion of Tregs defined by “CD25 and FOXP3 double positive” (a, c, j, n, o, p, q, t, ac) in PB of RA patients was lower than that of control subjects (REM -2.42, [-3.49 to -1.34]) (I^2^ = 96%, P < 0.0001) (Fig 4). Finally, when Tregs were defined by “CD25-high and FOXP3-positive” (b, m), the proportion of Tregs in PB of RA patients was significantly lower than that of control subjects (REM -1.03, [-1.42 to -0.63]) (S2D Fig). Heterogeneity assessed by I^2^ statics was 0% (P = 0.8443) suggesting very low heterogeneity.

### Disease activity of RA and the proportion of Tregs in PB of RA patients

**Fig 5:**
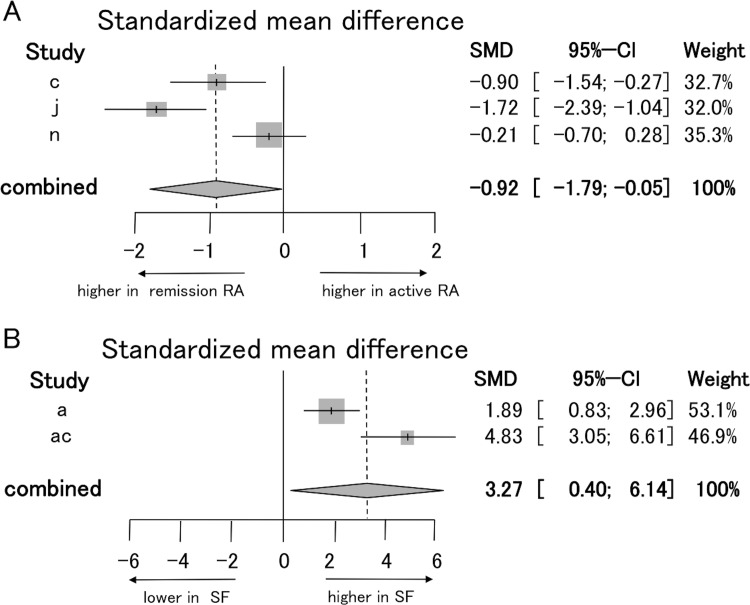
Forest plots of the proportion of Tregs in peripheral blood of different diseases activity and synovial fluid. (A) Standardized mean difference (the proportion of Tregs among CD4^+^ T cells in PB of active RA patients minus that of remission RA patients) was estimated by meta-analysis. Tregs, which were defined by “CD25 and FOXP3 double positive”, were selected for meta-analysis. (B) Standardized mean difference (the proportion of Tregs among CD4^+^ T cells in SF of RA patients minus that in PB of control subjects) was estimated by meta-analysis. Tregs, which were defined by “CD25 and FOXP3 double positive”, were selected for meta-analysis.

Whether the proportion of Tregs among CD4^+^ T cells in PB differed between active RA patients and remission RA patients was analyzed (S3 Table). Five studies (c, j, l, n, w) evaluated the proportion of Tregs in active RA and remission RA patients. Studies (l, w) could not be used in sub-analysis because the definitions of Tregs in these two studies were CD25^+^ alone and CD25-bright respectively and only one study per group is insufficient for meta-analysis. Three of these studies (c, j, n) in which Tregs were defined by “CD25 and FOXP3 double positive” were selected to perform the meta-analysis, revealing that there was a significant reduction in the proportion of Tregs in PB of active RA patients compared to remission RA patients (REM -0.92, [-1.79 to -0.05]) (Fig 5A). Heterogeneity assessed by I^2^ statics was 86.4% (P = 0.0007).

### The proportion of Tregs in synovial fluid of RA patients

The difference in the proportion of Tregs among CD4^+^ T cells of RA patients between SF and PB was evaluated (S4 Table). Five studies (a, f, k, ac, ae) evaluated the proportion of Tregs among CD4^+^ T cells in SF and PB of RA patients. Study (ac) provided data using three definitions of Tregs (CD25^+^, CD25-high, and CD25^+^ FOXP3^+^). To avoid the problem of variable definitions of Tregs, we did not use the data from two studies (ac, ae) in which Tregs were defined by CD25 positive alone in sub-analysis and instead focused on the definitions that previous research about Tregs and the results of our analysis of PBMC suggested are most reliable (CD25hi and CD25^+^ FOXP3^+^). Additionally, we avoided using studies (f, k, ac) in which Tregs were defined by CD25-high in sub-analysis because the CD25-high gating strategy in SF was not shown. Finally, two studies (a, ac) in which Tregs were defined as “CD25 and FOXP3 double positive” were selected for meta-analysis (REM 3.27, [0.40 to 6.14]) (Fig 5B); the results revealed that the proportion of Tregs among CD4^+^ T cells in SF was significantly higher than that in PB of RA patients. Heterogeneity assessed by I^2^ statics was 87% (P = 0.0056).

## Discussion and Conclusions

Functional deficiency of Tregs has been considered a cause of autoimmune diseases including RA. However, the function of Tregs in peripheral immune tolerance of RA patients was not elucidated sufficiently in previous researches. Even in the proportion of Tregs in PB of RA patients, a common consensus about it was not provided from previous reports. Therefore, the status of Tregs in RA patients is currently under debate. To elucidate the status of Tregs in RA, we performed meta-analysis of the proportion of Tregs among CD4^+^ T cells in RA patients. Meta-analysis of all selected studies revealed that there was no significant difference in the proportion of Tregs between RA patients and control subjects. Surprisingly, some studies showed quite different results. Some studies showed that the proportion of Tregs was higher in control and other studies showed that the proportion of Tregs was higher in RA. The major problem was considered to be inconsistent definitions of Tregs. Our sub-analysis showed that stricter definitions reduced the heterogeneity of results and consistently came to the same conclusion. The proportion of Tregs, when defined as “CD25-high”, “CD25-high and Foxp3 double positive” or “CD25 and FOXP3 double positive”, among CD4^+^ T cells in the PB of RA patients was decreased. The definition of “CD25 high and FOXP3 double positive” showed minimum heterogeneity.

CD25 is a well-defined marker of Tregs. However, the proportion of Tregs defined by CD25 expression alone was not clearly associated with the status of proper Tregs with suppressive function. One reason for this may be that the CD25 positive population contains FOXP3 negative effecter T cells [56]. Although loss of CD127 expression is often utilized to exclude effector T cells, the proportion of Tregs, which were defined by CD25^+^ and CD127 negative, the results were varied among selected studies in our research. Sakaguchi et al. reported that only the 1–2% of cells with the highest CD25 expression exert suppressive functions, and should therefore be considered as true Tregs [57]. To address this, some studies assess the proportion of CD25-high cells, however the consensus of accurate cutoff to define CD25-high has been not established. Therefore, we selected studies in which the CD25-high CD4^+^ T cell population was clearly defined and the proportion of CD25-high CD4^+^ defined Tregs in RA patients was lower than that in control subjects with slightly lower dispersion. This suggested that excluding cells expressing low levels of CD25 (probable effecter CD4^+^ T cells) was important for evaluation of Tregs.

It is well known that the *FOXP3* gene is a master regulator of Tregs and expression of FOXP3 is also utilized as a very important marker of Tregs. In our study, however, the proportion of Tregs, which were defined by FOXP3 positive alone, among CD4^+^ T cells in PB of RA patients was not significantly different to control subjects. The dispersion of results was extreme. Instead, the proportion of Tregs defined by “CD25 and FOXP3 double positive” was decreased (Fig 4) and the definition of Tregs by "CD25-high and FOXP3 positive" showed decreasing of the proportion of Tregs in PB and minimizing the heterogeneity of studies (S2D Fig) [26, 37]. From these results, more accurate definitions could give a uniform distribution of results.

Meanwhile, the proportion of Tregs in the SF of RA patients was increased in comparison to PB (Fig 5B). It seems likely that this is due to increased migration of Tregs to the SF in response to inflammation. The migration of Tregs is well known in carcinoma and infection [58–60]. Moreover, intravenous injected Tregs inhibited arthritic symptoms in CIA mouse [10]. Some parts of TCR repertoires of Tregs in SF with juvenile idiopathic arthritis (JIA) patients corresponded those of Tregs in PB with JIA patients [61]. These results indicate that Tregs, which derive from the thymus and circulate in PB, may migrate from PB to inflammatory tissue such as the synovium and then the proportion of Tregs in PB decreases and that of Tregs in SF increases. In addition to migration, FOXP3^+^ T cells are induced from naïve CD4^+^ T cells under inflammatory conditions such as the synovial tissue during arthritis [57, 62]. Both migration of PB Tregs to SF and conversion of non-Tregs to Tregs in SF should have enhanced regulatory function of autoimmunity. However, increased number of Tregs in the SF of RA patients was clearly not sufficient to prevent the autoimmunity. The suppressive function of Tregs in SF of RA patients has been demonstrated to be weak [35], partly because the suppressive function of Tregs migrating from PB may be impaired by TNF-α produced in SF [35, 63].

This study has several limitations. First, disease activity in RA patients was not consistent across studies. However, it is postulated that most studies selected RA patients with high disease activity. Second, disease duration from onset of RA was not consistent across studies. A previous study indicated that the proportion of Tregs in early stage of RA might be high and that in late stage of RA might be low [64]. In many of the selected studies, disease duration ranged from 5 to 15 years. Finally, RA treatments were not consistent across studies. These treatments may influence on the proportion of Tregs in RA patients. It is difficult to exclude the influence of RA treatments on our results.

Previously, Miyara et al. demonstrated the sub-population of FOXP3^+^ (or CD25^+^) Tregs in PB by utilizing CD45RA and FOXP3 (or CD25). They showed that CD45RA^+^ FOXP3 (or CD25)-low cells were naïve Tregs (Fraction [Fr.] 1); CD45RA^-^ FOXP3 (CD25)-high cells were effector Tregs (Fr. 2); and CD45RA^-^ FOXP3 (or CD25)-low cells were non-Tregs (Fr. 3) [18]. Accurate definitions of Tregs such as the Miyara Fractions may provide us more reliable information of the status of Tregs in human. In our selected studies, 2 studies utilized the Miyara Fraction model to define Tregs. In these 2 studies, the proportion of effector Tregs (Fr. 2) in RA patients was lower than that in control subjects [31, 37]. Function related definitions of Tregs such as the Miyara Fractions might become important in the future.

In conclusion, the lack of clarity of previous studies as to the perturbation of the status of Tregs in RA was caused by the lack of a consistent identification method for Tregs. Here we demonstrated that differences in the definitions of Tregs could influence on the results of the proportion of Tregs. Evaluation of Tregs by stricter methods such as “CD25hi” and “CD25 and FOXP3 double positive” consistently identify that the proportion of Tregs may be decreased in PB of RA patients. It is beyond the scope of our meta-analysis to identify which definitions of Tregs are correct, rather we can see which definitions provide consistent results. However, since stricter definitions of Tregs that have been validated by gold standard methods such as by analysis of suppressive activity and demethylation of FOXP3 CNS2 [18, 57] consistently identify that the proportion of Tregs in PB are reduced in RA patients, this is a strong indication that this may be the case. As a result, it is likely that if the proportion of Tregs differs in RA patients, accurate and functionally relevant definitions of Tregs are necessary to elucidate their status in RA.

## Supporting Information

S1 Fig Funnel plot for combined meta-analysis of the proportion of Tregs among CD4^+^ T cells in peripheral blood. Publication bias was evaluated by funnel plot. Each solid circle represents a study. The y-axis represents standard error that reflects the number of the samples, and the x-axis shows standard mean difference (the proportion of Tregs among CD4^+^ T cells in PB of RA patients minus that of control subjects) that reflects the effect size. Dotted line indicates random effect model estimate and dashed line indicates fixed effect model estimate. The outer dashed lines indicate the triangular region within which 95% of studies are expected to lie in the absence of both biases and heterogeneity.
(TIFF)

S2 Fig Forest plot for sub-group meta-analysis of the proportion of Tregs defined by specified definition. Standardized mean difference (the proportion of Tregs among CD4^+^ T cells in PB of RA patients minus that of control subjects) was estimated by meta-analysis. (A) Tregs, which were defined by “CD25 positive”, were analyzed. (B) Tregs, which were defined by “CD25 positive and CD127 negative”, were analyzed. (C) Tregs, which were defined by “FOXP3 positive”, were analyzed. (D) Tregs, which were defined by “CD25-high and FOXP3 positive”, were analyzed.
(TIFF)

S1 File PRISMA checklist. (DOC)

S1 Table Background of RA patients in each study. Patient characteristics of RA patients in each study. Studies (c, j, l, n, r) were shown information of Remission RA patients, too. ABT = abatacept; CRP = C-reactive protein; DAS28 = disease activity score 28; DMARDs = disease modified anti-rheumatic-drugs; ESR = erythrocyte sedimentation rate; iTNF = tumor necrosis factor-α inhibitor; MTX = methotrexate, NA = not applicable; CS = Corticosteroids; RA = rheumatoid arthritis; RF = rheumatoid factor, TCZ = tocilizumab.
(DOCX)

S2 Table The Newcastle–Ottawa Quality Assessment. The New Castle-Ottawa Assessment Scale. S1: case definition, S2: representativeness of the cases, S3: community controls, S4: no history of disease in controls, C1a: age-matched controls, C1b: controls for additional factor, E1: ascertainment of exposure (secure record), E1b: ascertainment of exposure (interview where blind to case/control status), E2: same method of ascertainment for case and controls, E3: non-response rate. (p) and (y) were not evaluated by The New Castle-Ottawa Assessment Scale in detail because these manuscripts were written in Chinese.
(DOCX)

S3 Table The proportion of Tregs in PB of active or remission RA patients. Studies (c, j, l, n, w) were shown information of % Tregs in PB among active RA and remission RA patients.
(DOCX)

S4 Table The proportion of Tregs in SF and PB of RA patients. Studies (a, f, k, ac, ae) were shown information of % Tregs in SF among RA patients.
(DOCX)
