# Supplementary figures and images for: The Proportion of Regulatory T Cells in Patients with Rheumatoid Arthritis: A Meta-Analysis
Source: PLoS One. 2016 Sep 13;11(9):e0162306. doi: 10.1371/journal.pone.0162306 (PMC5021283; doi:10.1371/journal.pone.0162306)

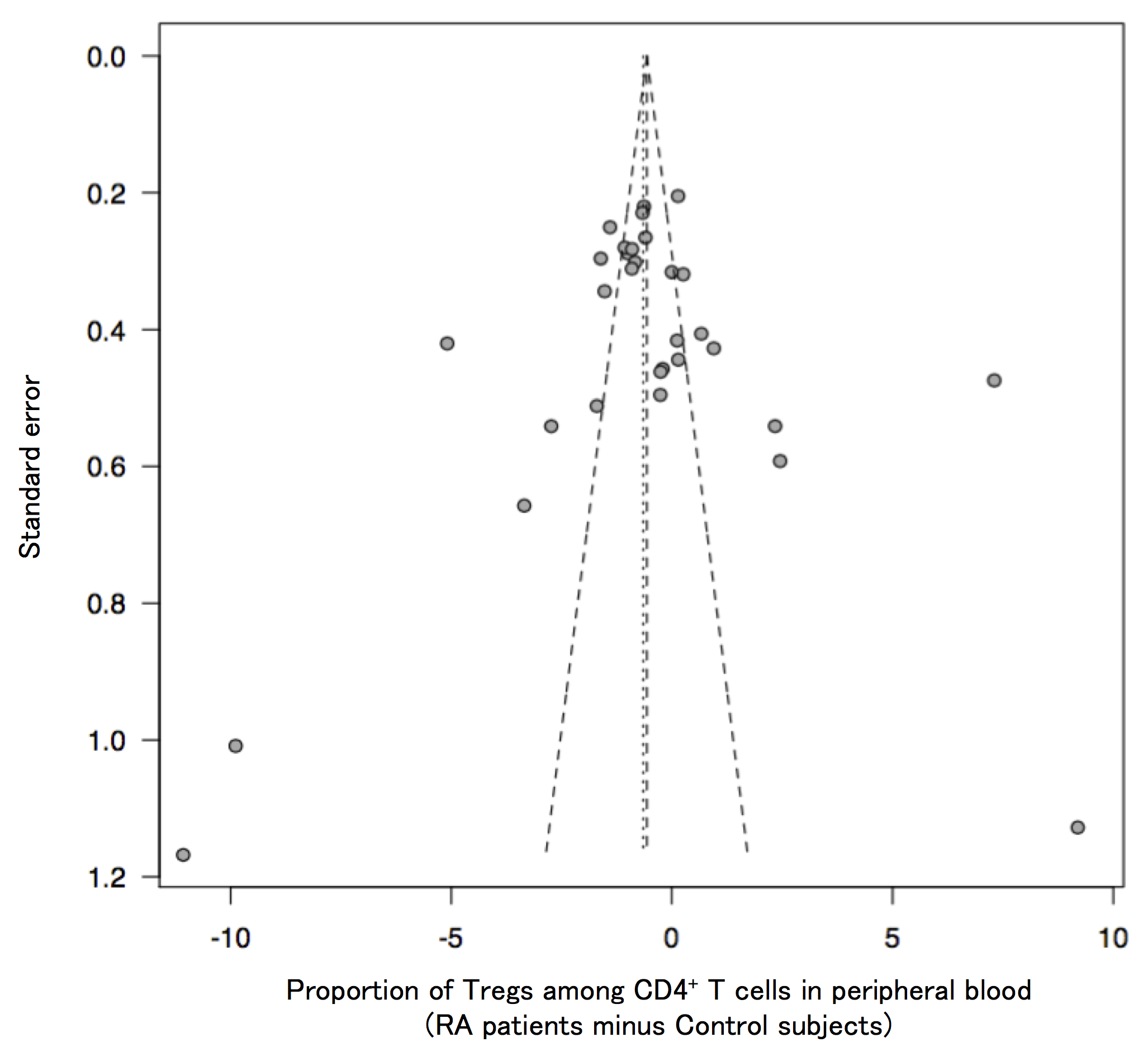

Supplement: S1 Fig — Publication bias was evaluated by funnel plot. Each solid circle represents a study. The y-axis represents standard error that reflects the number of the samples, and the x-axis shows standard mean difference (the proportion of Tregs among CD4+ T cells in PB of RA patients minus that of control subjects) that reflects the effect size. Dotted line indicates random effect model estimate and dashed line indicates fixed effect model estimate. The outer dashed lines indicate the triangular region within which 95% of studies are expected to lie in the absence of both biases and heterogeneity. (TIFF) [file pone.0162306.s001.tiff]

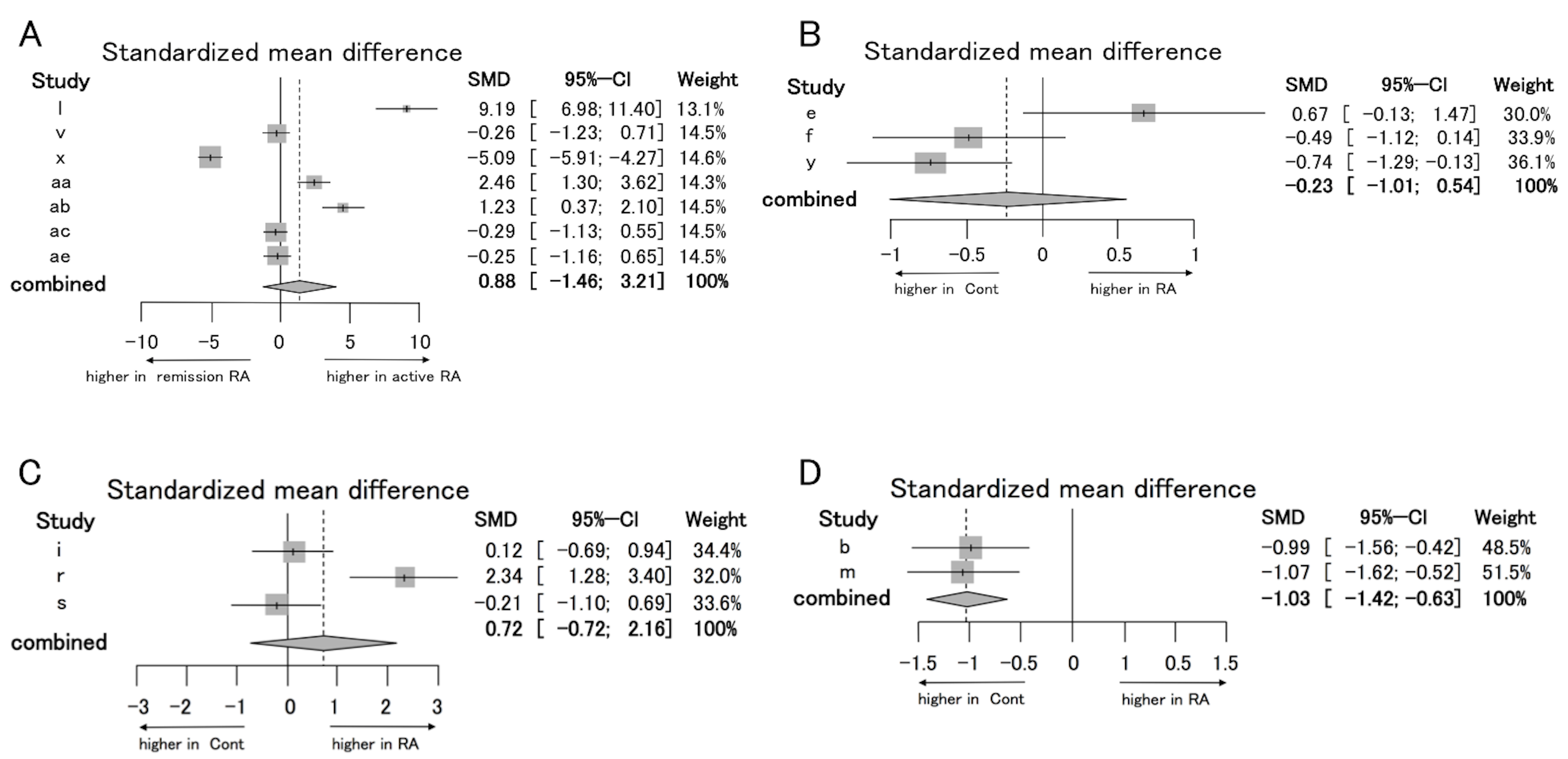

Supplement: S2 Fig — Standardized mean difference (the proportion of Tregs among CD4+ T cells in PB of RA patients minus that of control subjects) was estimated by meta-analysis. (A) Tregs, which were defined by “CD25 positive”, were analyzed. (B) Tregs, which were defined by “CD25 positive and CD127 negative”, were analyzed. (C) Tregs, which were defined by “FOXP3 positive”, were analyzed. (D) Tregs, which were defined by “CD25-high and FOXP3 positive”, were analyzed. (TIFF) [file pone.0162306.s002.tiff]
