# Supplementary material for: The Proportion of Regulatory T Cells in Patients with Rheumatoid Arthritis: A Meta-Analysis
Source: PLoS One. 2016 Sep 13;11(9):e0162306. doi: 10.1371/journal.pone.0162306 (PMC5021283; doi:10.1371/journal.pone.0162306)
Supplement: S1 Table — Patient characteristics of RA patients in each study. Studies (c, j, l, n, r) were shown information of Remission RA patients, too. ABT = abatacept; CRP = C-reactive protein; DAS28 = disease activity score 28; DMARDs = disease modified anti-rheumatic-drugs; ESR = erythrocyte sedimentation rate; iTNF = tumor necrosis factor-α inhibitor; MTX = methotrexate, NA = not applicable; CS = Corticosteroids; RA = rheumatoid arthritis; RF = rheumatoid factor, TCZ = tocilizumab. (DOCX) [file pone.0162306.s004.docx]

**S1 Table. Background of RA patients in each study**

| Author (Study number)  [Remission RA data] | Age (years)  (mean ± SD) | Female  (%) | Disease duration  (years) | RF positivity  (%) | DAS28 | CRP  (mg/dl) | ESR  (mm/hour) | Treatment |
| --- | --- | --- | --- | --- | --- | --- | --- | --- |
| (a) Barbieri A. et al. [25] | 54 ± 16 | 71 | NA | NA | NA | NA | NA | NA |
| (b) Al-Zifzafa DS. et al. [26]. | 40 ± 7.7 | 83 | 4.6 ± 3.1 | NA | 4.6 ± 1.2 | 1.0 ± 1.3 | 49 ± 20 | NA |
| (c) Gaafar T. et al. [27]  [Remission RA data] | 49 ± 12  36 ± 12 | NA  NA | (1 - 28)  NA | 61  NA | 7.0 ± 0.7  2.9 ± 0.5 | 2.4 ± 0.6  NA | NA  NA | NA  NA |
| (d) Daïen IC. et al. [28] | 59 ± 13 | 72 | 14 ± 12 | 76 | 4.6 ± 1.4 | 0.2 ± 0.2 | NA | CS, DMARDs, iTNF, TCZ |
| (e) Cribbs AP. et al. [29]. | 52 ± 13 | 83 | 0.8 ± 0.7 | 78 | 5.5 ± 0.8 | NA | NA | NA |
| (f) Moradi B. et al. [30] | 68 ± 6.0 | 39 | 14 ± 0.7 | 56 | 4.5 ± 1.2 | 1.7 ± 0.9 | 23 ± 12 | CS, DMARDs, iTNF |
| (g) Matsuki F. et al. [31] | 46 ± 6.7 | 81 | 7.1 ± 7.6 | NA | 3.1 ± 1.3 | NA | NA | CS, DMARDs, iTNF, ABT |
| (h) G. Guggino et al. [32] | 44 ± 3.0 | 80 | 1.0 ± 0.6 | 72 | 6.0 ± 1.6 | 0.5 ± 0.0 | 57 ± 22 | CS, DMARDs |
| (i) Ji L. et al. [33] | 51 ± 14 | 81 | 5.3 ± 6.3 | NA | 4.6 ± 1.5 | NA | NA | CS, DMARDs, Biologics |
| (j) Gao S. et al. [34]  [Remission RA data] | 53 ± 2.3  50 ± 12 | 88  78 | 5.9 ± 2.3  4.7 ± 2.2 | 88  72 | 5.0 ± 0.6  2.5 ± 0.4 | 6.8 ± 2.9  0.9 ± 0.7 | 77 ± 17  19 ± 10 | NA  NA |
| (k) Nie H. et al. [35] | 56 ± 11 | 89 | 11 ± 8.6 | 90 | 5.3 ± 1.4 | 3.8 ± 3.8 | 54 ± 35 | DMARDs |
| (l) Abazaa N. et al. [36]  [Remission RA data] | 44 ± 6.9  41 ± 7.8 | 90  85 | 6.8 ± 4.6  5.4 ± 3.29 | NA  NA | 5.2 ± 1.0  1.9 ± 0.5 | 24 ± 9.8  4.4 ± 1.3 | 52 ± 19  9.7 ± 2.7 | MTX  NA |
| (m) Kim JR. et al. [37] | 57 ± 13 | 26 | NA | NA | NA | NA | NA | NA |
| (n) Niu Q. et al. [38]  [Remission RA data] | 48 ± 12  44 ± 9.0 | 17  17 | NA  NA | NA  NA | NA  NA | NA  NA | NA  NA | NA  NA |
| (o) Chen J. et al. [39] | NA | NA | NA | NA | NA | NA | NA | NA |
| (p) Chen R. et al. [40] | NA | NA | NA | NA | NA | NA | NA | NA |
| (q) Xiao H. et al. [41] | 55 ± NA | 80 | 11 ± 8.2 | NA | NA | 1.5 ± 0.8 | 45 ± 29 | CS, MTX, iTNF |
| (r) Furuzawa-Carballeda J. et al. [42]  [Remission RA data] | 53 ± 14  44 ± 11 | 100  100 | 16 ± 10  13 ± 7.5 | NA  NA | NA  NA | NA  NA | 34 ± 17  20 ± 12 | CS, DMARDs  NA |
| (s) Loza MJ. et al. [43] | (36 - 68) | NA | NA | NA | NA | NA | NA | NA |
| (t) Tang TT. et al. [44] | 51 ± 13 | 71 | 12 ± 8.4 | 71 | 5.7 ± 0.9 | 1.4 ± 0.8 | 21 ± 8.1 | CS, DMARDs, iTNF |
| (u) Chen MH. Et al. [45] | 57 ± 2.4 | 70 | 8.0 ± 1.8 | NA | 3.1 ± 0.3 | 1.2 ± 0.7 | 26 ± 6.2 | CS, MTX, DMARDs, iTNF |
| (v) Ursaciuc C. et al. [46] | NA | NA | NA | NA | NA | NA | NA | NA |
| (w) Al-Shukaili A. et al. [47] | 45 ± 15 | 83 | NA | NA | 4.7 ± NA | NA | NA | NA |
| (x) Sempere-Ortells JM. et al. [48] | 58 ± 9.0 | 67 | 11 ± 8.1 | 64 | 3.7 ± 1.0 | 11 ± 1.8 | 25 ± 11 | MTX, iTNF |
| (y) Huang ZX. et al. [49] | 33 ± 5.0 | 56 | NA | NA | 6.3 ± 0.5 | 3.4 ± 1.8 | 49 ± 11 | NA |
| (z) Han GM. et al. [50] | 46 ± 11 | 82 | 6 ± NA | 64 | NA | 3.2 ± 2.8 | 44 ± 30 | CS, DMARDs |
| (aa) Yoon BY. et al. [51] | 51 ± 12 | 67 | 7.3 ± 5.7 | 67 | NA | 1.7 ± 1.9 | 27 ± 22 | PSL, MTX |
| (ab) Kao JK. et al. [52] | 59 ± 12 | 79 | NA | 83 | 5.9 ± 0.7 | NA | NA | NA |
| (ac) Jiao Z. et al. [53] | NA | NA | NA | NA | NA | NA | NA | NA |
| (ad) Minami R. et al. [54] | 59 ± 13 | 82 | NA | NA | NA | NA | NA | NA |
| (ae) Möttönen M. et al. [55]. | 56 ± NA | NA | 14 ± NA | NA | NA | NA | NA | CS, DMARDs |

Patient characteristics of RA patients in each study. Studies (c, j, l, n, r) were shown information of Remission RA patients, too. ABT = abatacept; CRP = C-reactive protein; DAS28 = disease activity score 28; DMARDs = disease modified anti-rheumatic-drugs; ESR = erythrocyte sedimentation rate; iTNF = tumor necrosis factor-α inhibitor; MTX = methotrexate, NA = not applicable; CS = Corticosteroids; RA = rheumatoid arthritis; RF = rheumatoid factor; TCZ = tocilizumab.
