# Supplementary material for: The Proportion of Regulatory T Cells in Patients with Rheumatoid Arthritis: A Meta-Analysis
Source: PLoS One. 2016 Sep 13;11(9):e0162306. doi: 10.1371/journal.pone.0162306 (PMC5021283; doi:10.1371/journal.pone.0162306)
Supplement: S2 Table — The New Castle-Ottawa Assessment Scale. S1: case definition, S2: representativeness of the cases, S3: community controls, S4: no history of disease in controls, C1a: age-matched controls, C1b: controls for additional factor, E1: ascertainment of exposure (secure record), E1b: ascertainment of exposure (interview where blind to case/control status), E2: same method of ascertainment for case and controls, E3: non-response rate. (p) and (y) were not evaluated by The New Castle-Ottawa Assessment Scale in detail because these manuscripts were written in Chinese. (DOCX) [file pone.0162306.s005.docx]

**S2 Table.** The Newcastle–Ottawa Quality Assessment

| Author (Study number) | S1 | S2 | S3 | S4 | C1a | C1b | E1a | E1b | E2 | E3 |
| --- | --- | --- | --- | --- | --- | --- | --- | --- | --- | --- |
| (a) Barbieri A. et al. [25] | * |  |  |  |  |  | * |  | * |  |
| (b) Al-Zifzafa DS. et al. [26]. | * | * | * | * | * |  | * |  | * |  |
| (c) Gaafar T. et al. [27] | * | * | * | * | * |  | * |  | * |  |
| (d) Daïen IC. et al. [28] | * | * | * |  | * |  | * |  | * |  |
| (e) Cribbs AP. et al. [29]. | * |  | * | * | * |  | * |  | * |  |
| (f) Moradi B. et al. [30] | * | * |  |  | * |  | * |  | * |  |
| (g) Matsuki F. et al. [31] | * |  |  | * | * |  | * |  | * |  |
| (h) G. Guggino et al. [32] | * | * | * | * | * |  | * |  | * |  |
| (i) Ji L. et al. [33] | * | * | * | * | * |  | * |  | * |  |
| (j) Gao S. et al. [34] | * |  | * | * |  |  | * |  | * |  |
| (k) Nie H. et al. [35] | * | * | * | * | * |  | * |  | * |  |
| (l) Abazaa N. et al. [36] | * | * | * | * | * |  | * |  | * |  |
| (m) Kim JR. et al. [37] | * |  | * | * | * |  | * |  | * |  |
| (n) Niu Q. et al. [38] | * |  | * | * | * |  | * |  | * |  |
| (o) Chen J. et al. [39] | * | * |  |  |  |  | * |  | * |  |
| (p) Chen R. et al. [40] | - | - | - | - | - | - | - | - | - | - |
| (q) Xiao H. et al. [41] | * |  | * | * | * |  | * |  | * |  |
| (r) Furuzawa-Carballeda J. et al. [42] | * | * | * | * | * |  | * |  | * |  |
| (s) Loza MJ. et al. [43] | * |  | * | * |  |  | * |  | * |  |
| (t) Tang TT. et al. [44] | * | * |  |  | * |  | * |  | * |  |
| (u) Chen MH. Et al. [45] | * | * |  |  |  |  | * |  | * |  |
| (v) Ursaciuc C. et al. [46] | * |  |  | * |  |  | * |  | * |  |
| (w) Al-Shukaili A. et al. [47] | * |  | * | * |  |  | * |  | * |  |
| (x) Sempere-Ortells JM. et al. [48] | * | * | * | * | * |  | * |  | * |  |
| (y) Huang ZX. et al. [49] | - | - | - | - | - | - | - | - | - | - |
| (z) Han GM. et al. [50] | * | * | * | * | * |  | * |  | * |  |
| (aa) Yoon BY. et al. [51] | * | * | * | * |  |  | * |  | * |  |
| (ab) Kao JK. et al. [52] | * | * | * | * | * |  | * |  | * |  |
| (ac) Jiao Z. et al. [53] | * | * | * | * | * |  | * |  | * |  |
| (ad) Minami R. et al. [54] | * |  |  |  |  |  | * |  | * |  |
| (ae) Möttönen M. et al. [55]. | * |  | * | * | * |  | * |  | * |  |

The New Castle-Ottawa Assessment Scale. S1: case definition, S2: representativeness of the cases, S3: community controls, S4: no history of disease in controls, C1a: age-matched controls, C1b: controls for additional factor, E1: ascertainment of exposure (secure record), E1b: ascertainment of exposure (interview where blind to case/control status), E2: same method of ascertainment for case and controls, E3: non-response rate. (p) and (y) were not evaluated by The New Castle-Ottawa Assessment Scale in detail because these manuscripts were written in Chinese.
