# Supplementary material for: The Proportion of Regulatory T Cells in Patients with Rheumatoid Arthritis: A Meta-Analysis
Source: PLoS One. 2016 Sep 13;11(9):e0162306. doi: 10.1371/journal.pone.0162306 (PMC5021283; doi:10.1371/journal.pone.0162306)
Supplement: S3 Table — Studies (c, j, l, n, w) were shown information of % Tregs in PB among active RA and remission RA patients. (DOCX) [file pone.0162306.s006.docx]

**S3 Table. The proportion of Tregs in PB of active or remission RA patients**

| **Author [Ref.]** | **Numbers** | **% of Tregs among CD4^+^ T cells (mean/median ± SD)** |
| --- | --- | --- |
| (c) Gaafar T. et al. [27] | active RA: 38, remission RA: 14 | active RA: 1.01 ± 0.87, remission RA: 1.80 ± 0.83 |
| (j) Gao S. et al. [34] | active RA: 35, remission RA: 17 | active RA: 2.89 ± 0.17, remission RA: 3.21 ± 0.21 |
| (l) Abazaa N. et al. [36] | active RA: 20, remission RA: 20 | active RA: 19.7 ± 0.57, remission RA: 19.0 ± 0.53 |
| (n) Niu Q. et al. [38] | active RA: 36, remission RA: 30 | active RA: 1.54 ± 0.23, remission RA: 1.59 ± 0.24 |
| (w) Al-Shukaili A. et al. [47] | active RA: 10, remission RA: 20 | active RA: 0.56 ± 0.29, remission RA: 1.83 ± 0.76 |

Studies (c, j, l, n, w) were shown information of % Tregs in PB among active RA and remission RA patients. PB = peripheral blood; SD = standard deviation; Ref. = reference.
