# Supplementary material for: The Proportion of Regulatory T Cells in Patients with Rheumatoid Arthritis: A Meta-Analysis
Source: PLoS One. 2016 Sep 13;11(9):e0162306. doi: 10.1371/journal.pone.0162306 (PMC5021283; doi:10.1371/journal.pone.0162306)
Supplement: S4 Table — Studies (a, f, k, ac, ae) were shown information of % Tregs in SF among RA patients. (DOCX) [file pone.0162306.s007.docx]

**S4 Table. The proportion of Tregs in SF and PB of RA patients.**

| **Author [Ref.]** | **Numbers** | **Tregs definition** | **% of Tregs among CD4^+^ T cells**  **(mean/median±SD)** |
| --- | --- | --- | --- |
| (a) Barbieri A. et al. [25] | RA: 14 | CD25+ FOXP3+ | PB: 5.0 ± 3.8, SF: 13.8 ± 4.8 |
| (f) Moradi B. et al. [30] | RA: 18 | CD25-high  CD25+ CD127- | PB: 4.9 ± 2.0, SF: 10.0 ± 6.3  PB: 6.7 ± 1.8, SF: 11.8 ± 5.5 |
| (k) Nie H. et al. [35] | RA: 20 | CD25-high | PB: 1.7 ± 0.9, SF: 1.9 ± 0.9 |
| (ac) Jiao Z. et al. [53] | RA: 11 | CD25+  CD25-high  CD25+ FOXP3+ | PB: 10.0 ± 3.4, SF: 32.5 ± 5.2  PB: 2.3 ± 0.9, SF: 16.1 ± 4.7  PB: 3.1 ± 1.5, SF: 19.6 ± 4.4 |
| (ae) Möttönen M. et al. [55] | RA: 10 | CD25+ | PB: 5.5 ± 4.1, SF: 12.0 ± 4.8 |

Studies (a, f, k, ac, ad) were shown information of % Tregs in SF among RA patients. PB = peripheral blood; SD = standard deviation; Ref. = reference; SF = synovial fluid.
